# Progressive attenuation of visual global precedence across healthy aging and Alzheimer’s disease

**DOI:** 10.3389/fnagi.2022.893818

**Published:** 2022-09-20

**Authors:** Andrea Álvarez-San Millán, Jaime Iglesias, Anahí Gutkin, Ela I. Olivares

**Affiliations:** ^1^Department of Psychology, Faculty of Biomedical and Health Sciences, Universidad Europea de Madrid, Madrid, Spain; ^2^Department of Biological and Health Psychology, Faculty of Psychology, Universidad Autónoma de Madrid, Madrid, Spain; ^3^Department of Social Psychology and Methodology, Faculty of Psychology, Universidad Autónoma de Madrid, Madrid, Spain

**Keywords:** aging, Alzheimer’s disease, global precedence effect, global/local processing, longitudinal study, mild cognitive impairment, Navon, visual perception

## Abstract

In the perception of Navon hierarchical stimuli (e.g., large letters made up of small letters), young adults identify large letters faster than small ones (known as ‘global advantage’) and identify more slowly small letters when they form a different (or incongruent) large letter (known as ‘unidirectional global interference’). Since some global/local perceptual alterations might be occurring with aging, we investigated whether these effects vary across healthy aging and Alzheimer’s disease (AD). Here, the Navon letter task was administered to 26 healthy elderly (HE), 21 adults with mild cognitive impairment (MCI), and 26 adults with AD. The same task was administered 1 year later, and different neuropsychological variables were incorporated into the analyses. The cross-sectional study revealed no global advantage but did reveal both global and local interferences in all groups when response times were analyzed. Regarding discrimination sensitivity, HE showed unidirectional global interference, while AD displayed better discrimination of local than global letters in the incongruent condition, which denotes less interference by global distractors than by local ones. The longitudinal study revealed that 1 year later the participants with MCI showed a slowdown in inhibiting local distractors in the global task, revealing a certain bias toward focus in their attention on small stimuli. The elders with AD reflected a generalized slowing of their responses with a clear bias toward local analysis of stimuli, also suggested by their better discrimination in the incongruent local task at the second moment of assessment. Furthermore, all response timing measures in the Navon task were correlated with several neuropsychological indexes of highly sensitive neuropsychological tests, suggesting that performance in this task may also have a potential diagnostic value for differentiating typical from atypical cognitive aging. All these results support the need for a multidomain approach to define neuropsychological markers of progression toward AD, including visual perceptual organization evaluated *via* measures of performance quality.

## Introduction

Pathological aging entails alterations in one or more cognitive domains, and a recurrent aim of neuropsychological research is to define early markers of different age-related neurodegenerative disorders. Interestingly, people with Alzheimer’s disease (AD), besides from suffering the usual progressive impairment in the domains of memory, executive functions and language, also show deficits in visual perception (Adlington et al., 2009). For example, relevant changes in perceptual organization based on spatial relationships have been observed in older adults with AD vs. healthy older people, especially in perceptual grouping (Kurylo et al., 2003). Perceptual grouping is a mechanism considered an intermediate level of visual processing, based on the classical principles of proximity, good continuation, common fate, similarity, closure, symmetry and parallelism (Wertheimer, 1923; Wagemans et al., 2012). Grouping involves processes such as identification of fragmented figures, figure-ground separation, and integration of visual elements into a whole, the so-called global/local visual processing. Indeed, this mechanism precedes high-order visual functions, being necessary for face recognition and analysis of complex scenes (Kurylo et al., 2017). Perceptual grouping may also be impaired in healthy older adults (Lithfous et al., 2016). This could be associated in aging with faster and more pronounced atrophy of the right (vs. left) hemisphere (Dolcos et al., 2002), which is thought to be specialized in the holistic processing of complex stimuli (Fink et al., 1997).

An experimental task commonly performed to assess global/local visual processing was designed by Navon (1977) and consisted of the presentation of hierarchical letters (originally the large letters H and S formed by the small letters _H_ or _S_), so that participants must identify the large letter (i.e., the global level) or the small letters that make it up (i.e., the local level) in different blocks. The global and the local letters could be either congruent (e.g., global S and local _S_) or incongruent (e.g., global H and local _S_). Navon observed in young adults the global precedence effect (GPE), characterized by a global advantage (i.e., faster detection of the global vs. the local form) and by unidirectional global interference (i.e., slower analysis of the local level when irrelevant information from the global level is incongruent).

Several authors have analyzed the age-related effect on global/local visual processing obtaining very mixed results with different versions of the Navon task. Some studies have observed a facilitation of global processing in elderly typical adults similar to the one defined in young adults (Bruyer and Scailquin, 2000; Roux and Ceccaldi, 2001; Bruyer et al., 2003; Georgiou-Karistianis et al., 2006; Agnew et al., 2016; Bouhassoun et al., 2022), while others have identified an attenuation of global facilitation with increasing age (Müller-Oehring et al., 2007). In this regard, Filoteo et al. (1992, 1995), using numbers as stimuli in both direct and divided tasks (i.e., the latter requiring attention to the global and local levels simultaneously), and Staudinger et al. (2011), presenting letters as stimuli in a divided attention task, found no differences in healthy older participants between global vs. local identifications. The latter authors concluded that the GPE observed in young people disappears in older adults. Some authors have even observed a predisposition to local analysis in older adults (Insch et al., 2012), reflected in faster identifications of the local vs. the global form of the hierarchical stimuli presented (Coslett et al., 1995; Oken et al., 1999; Slavin et al., 2002; Lux et al., 2008; Lithfous et al., 2016).

There are several methodological differences between the studies cited, with those relating to exposure time and spatial location of the hierarchical stimuli being particularly striking. Regarding the exposure time, it has been shown that long exposures lead to attenuation of the GPE, affecting interference more than global advantage (Paquet and Merikle, 1984). In this regard, there is an important lack of systematization in the studies conducted with older adults, as the results found with very short exposure times of the stimuli (50 ms; Bruyer and Scailquin, 2000) are hardly comparable with those obtained with unlimited exposure time (i.e., up to response; Agnew et al., 2016; Bouhassoun et al., 2022), the latter allowing for slower exploration of the visual scene. Also, the spatial location of the hierarchical stimuli differs markedly between the different studies reviewed: their presentation has been in some studies fixed in the center of the screen and in others randomized in peripheral positions (e.g., in the different quadrants) of the screen. This is not a trivial issue, since it is also proved that the GPE becomes less likely when stimuli are presented centrally (Kimchi, 2015). That is, spatial certainty facilitates the focusing on local elements when they are centered in the foveal vision, as opposed to peripheral randomized presentation, which requires constant readjustment of the attentional focus. This factor is also key to explaining many of the discrepancies among results found in studies with older adults, since authors reported an absence of differences in older adults between global and local visual processing (Filoteo et al., 1992, 1995; Staudinger et al., 2011) or a facilitation of local processing (Slavin et al., 2002; Lux et al., 2008; Lithfous et al., 2016) based on results obtained by central presentation of stimuli; whereas in studies where the stimulus presentation was randomized across quadrants of the screen, a global facilitation was found (Bruyer and Scailquin, 2000; Roux and Ceccaldi, 2001; Bruyer et al., 2003; Agnew et al., 2016). On the other hand, the disparity in the average age of the studied groups of elderly participants could also explain to some extent the different results found by different authors. In this regard, all studies that observed a clear bias toward global analysis had an average age of 70 years or younger (Bruyer and Scailquin, 2000; Roux and Ceccaldi, 2001; Bruyer et al., 2003; Georgiou-Karistianis et al., 2006; Agnew et al., 2016; Bouhassoun et al., 2022), while studies that revealed a bias toward local analysis reported different mean ages, with the lowest being 58 years old (Lux et al., 2008) and the highest being 84 (Oken et al., 1999), i.e., with a wider age range including octogenarian adults.

Global/local visual processing in older adults with neurodegenerative disorders has also been the subject of some research, particularly in AD. In contrast to typical aging, which involves atrophy in forebrain areas affecting cortico-cortical connections between fore- and posterior regions, older adults with AD also show lesions in limbic structures such as the hippocampus and entorhinal cortex (Braak et al., 1999). Regarding visuoperceptual functions, a reduction in attentional focus has been reported in older adults with AD, evidenced by a greater predisposition to analyze the local elements (vs. the global form) of a visual scene (Matsumoto et al., 2000). In contrast to Filoteo et al. (1992, 1995), who found no global or local facilitation bias when presenting numbers as hierarchical stimuli, several authors have observed a faster and more effective analysis of the local level compared to the global level in older people with AD using global/local visual processing tasks (Coslett et al., 1995; Slavin et al., 2002; Blanca et al., 2010). This suggests that older people with AD, unlike typical older adults, are characterized by marked deficits in the global processing of complex visual stimuli.

Intriguingly, there is a lack of studies on prodromal stages of AD in this field of research. Several studies have documented in a number of cases an accelerated rate of progression to AD from an MCI condition, such a condition being considered a prodromal stage of Alzheimer’s type dementia (Albert et al., 2011), in which non-age-related impairments in one or several cognitive functions emerge without substantially interfering with activities of daily living (Petersen et al., 1999; Petersen, 2004). Patients with MCI suffer from neuromorphological changes in addition to those associated with age, characterized by gray matter reductions in the hippocampus, entorhinal cortex, and cingulate gyrus (Yang et al., 2012). It is therefore of particular interest to investigate global/local visual processing in older adults with MCI to identify early markers of visual perception deficits associated with cognitive decline and to try to determine at what point changes that might be observed in AD in relation to visual processing of complex visual objects become apparent.

In order to find early neurocognitive signatures of global/local visual processing in pathological aging, in the present study, we carried out two complementary studies on the evolution of global/local visual processing across typical aging and AD. First, we conducted a cross-sectional study with healthy elderly (HE) and elderly people with mild cognitive impairment (MCI) and AD. Second, we conducted a longitudinal study 1 year later in which we assessed global/local visual processing in relation to several indexes of neuropsychological functioning sensitive to cognitive decline. This included a comparison between patients with MCI who did not change diagnosis vs. those who at the second time of assessment had been diagnosed with AD. Our participants performed a Navon letter task in which stimuli were randomly displayed in different quadrants of the screen for 500 ms. Moreover, our experimental design considered methodological issues that have limited the scope of findings in previous literature, including for the first time the analysis of elderly participants in the prodromal stage of Alzheimer’ type dementia. We hypothesized that the global/local visual processing in our sample would be characterized by attenuation of global facilitation in participants with MCI as compared to participants with HE which in turn would be less accentuated than that observed in participants with AD.

## Study 1: A cross-sectional study on differences in global/local visual processing in healthy elderly, mild cognitive impairment, and Alzheimer’s disease

### Methods

#### Participants

The study included three groups of elderly participants, namely, one each of HE, MCI, and AD. All the subjects or their legal guardians (usually a family member) signed written informed consent forms, and the university ethics committee approved the study (code: CEI-71-1271).

The elders were residents and attendees of day centers for the elderly in the Autonomous Community of Madrid. The Global Deterioration Scale - GDS (Reisberg et al., 1982), the Mini-cognitive Examination 30-item version - MEC-30 (Lobo et al., 1999, validated Spanish version of the Mini-Mental Status Examination - MMSE, Folstein et al., 1975), the abbreviated Yesavage questionnaire (Sheikh and Yesavage, 1986), the Neuropsychiatric Inventory – NPI (Cummings, 1997) and the Edinburgh Handedness Inventory (Oldfield, 1971) were administered. Exclusion criteria were severe cognitive impairment (MEC-30 < 10 points), uncorrected vision problems (i.e., blindness, cataract, glaucoma, retinal detachment, etc.), language production problems, history of neurological disorders (i.e., epilepsy, strokes, and neurodegenerative disorders other than AD), depressive symptomatology (i.e., participants with diagnosis of major depression or with a score of 10 points or more in the Geriatric Depressive Scale - Sheikh and Yesavage, 1986), major psychiatric disorder and alcohol or substance abuse.

Table 1 shows the characteristics of the 73 participants: 26 HE, 21 MCI, and 26 AD. The psychogeriatricians at each center who, in addition to daily follow-up, administered a brief cognitive and affective assessment every 6 months (using the GDS, MEC-30, Yesavage abbreviated questionnaire, and the NPI), established the diagnoses based on the MCI criteria described by Petersen et al. (1999) and Petersen (2004) and according to the criteria for ‘Alzheimer’s clinical syndrome’ (previously termed as ‘probable AD,’ Mckhann et al., 2011) established by the National Institute on Aging-Alzheimer’s Association (NIA-AA, Jack et al., 2018). The research framework recently promoted by the NIA-AA (Jack et al., 2018) considers cognitive performance in aging as a continuum of three stages, namely, cognitively unimpaired, mild cognitive impairment and dementia. The present study was based on this research framework.

**TABLE 1:** Characteristics of the groups studied in the cross-sectional study.

	HE (*n* = 26)	MCI (*n* = 21)	AD (*n* = 26)	*F or* χ ^2^ (*df*)	*p* (ηp^2^)
Age	85.5 (5.31)	88.5 (7.00)	85.3 (1.16)	1.90 (2, 70)	0.16
Sex (M:F)	12 : 14	7 : 14	10 : 16	0.82 (2)	0.66
Education (I/II/III/IV)	(0/17/3/6)	(1/18/0/2)	(2/21/2/1)	7.33 (2)	**0.026 (0.09)**
Handedness	1.15 (0.25)	1.19 (0.39)	1.16 (0.44)	1.52 (2)	0.47
MMSE^a^	28.5 (1.33)	25.3 (3.24)	19.2 (3.40)	71.9 (2, 51)	<0.001* (0.74)
GDS	1.12 (0.33)	2.00 (0.77)	3.31 (0.68)	53 (2)	<0.001* (0.43)

Sex: M, male and F, female. Level of education: I, no schooling; II, elementary school; III, secondary school; and IV, graduate school. MMSE, Spanish version of the Mini-Mental Status Examination; GDS, Global Deterioration Scale; HE, healthy elderly; MCI, mild cognitive impairment; and AD, Alzheimer’s disease. Handedness ranges from 1 (completely right-handed) to 5 (completely left-handed).

^a^Levene’s test indicated unequal variances *F*(2,70) = 7.81, *p* = 0.001, so degrees of freedom were adjusted by Brown–Forsythe, and Games–Howell *post hoc* pairwise comparison test was performed.

*Statistically significant differences (*p* < 0.05) are highlighted in bold.

Benzodiazepines were used in 11.5% of the HEs, 28.6% of the elders with MCI, and 23.1% of those with AD. In the latter case, 30.8% were also under treatment with donepezil (a specific and reversible cholinesterase inhibitor), two of them were in combination therapy with memantine (an antagonist of the *N*-methyl-D-aspartate -NMDA- receptor).

The three groups of participants (HE, MCI, and AD) were found to be homogeneous on the variables age, sex, and handedness (cf. Table 1). Regarding educational level, although differences were found (*p* = 0.026), Bonferroni-corrected pairwise comparisons revealed no significant differences between groups (HE vs. MCI *p* = 0.11; HE vs. AD *p* = 0.058; MCI vs. AD *p* = 0.99). The three groups did differ in their MMSE and GDS scores, reflecting worse performance on the MMSE and higher scores on the GDS scale through the HE, MCI, and AD groups.

#### Materials

The task was carried out individually in an acoustically insulated and well-illuminated room, and the participants were seated at a distance to the computer screen of approximately 70 cm. The stimuli were presented in black (3.19 cd/m^2^) on a white background (192.8 cd/m^2^) on 16-inch screens using the software ‘Estimulador Cognitivo’ (Neuronic S.A.).

The Navon letter task was administered with the large letters H and S, formed by small letters _H_ or _S_. The large letters measured 9 cm in height and 6.5 cm in width (vertical and horizontal visual angles 7.36° × 5.32°, respectively), while the small letters were 1.3 cm high and 1 cm wide (vertical and horizontal visual angles 1.06° × 0.82°, respectively). In order for the hierarchical letters to maintain the same size and dispersion, it was necessary to vary slightly the number of elements composing each letter, with the letter H being composed of 12 elements and the letter S of 14 elements.

**FIGURE 1:**
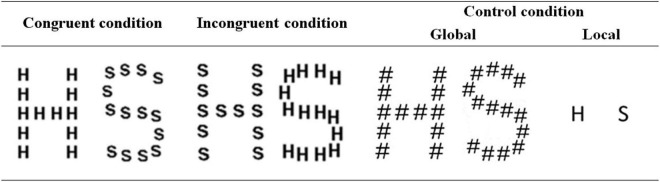
Stimuli presented in each condition for the global/local Navon identification task.

There were three experimental conditions for the presentation of the hierarchical letters: the congruent condition, when the same letter was presented as a large letter and small letters; the incongruent condition, when the large letter and small letters were different; and the control condition, when only a large or a small letter was visible depending on whether a global or a local directed attention task was performed. The control stimuli in the global task were the large letters H and S composed of the symbol # (which has no alphabetic meaning for native European Spanish people), while the control stimuli for the local task were the letters H and S presented singly in the same size as the elements used for the hierarchical figures. The stimuli and different experimental conditions are exemplified in Figure 1.

#### Procedure

Considering the mean age (∼87) of our participants, we have used a block design with directed attention to global or local stimuli. We ruled out divided attention tasks because they become more difficult to perform as people age (Künstler et al., 2018). Moreover, results derived from studies with such tasks with younger people (i.e., 55–70) using similar stimuli are still inconclusive (Filoteo et al., 1992, 1995; Lux et al., 2008; Staudinger et al., 2011). In our directed attention task, similar to other studies with older adults (Bruyer and Scailquin, 2000; Roux and Ceccaldi, 2001; Bruyer et al., 2003; Agnew et al., 2016), stimuli were administered in two blocks, one time asking the participant to attend selectively to the large letter (global level/task) and the other time to attend selectively to the small letter (local level/task). The participants were asked to respond as quickly and accurately as possible whether the letter present on the screen at the attended level was H or S. The participants performed 72 trials per block, except for 16 who performed 60 trials per task. This reduction in the number of trials was intended to avoid distraction and fatigue of the participants during the task, with the number of trials administered to healthy and cognitively impaired participants being similar to that employed by other authors (Slavin et al., 2002; Georgiou-Karistianis et al., 2006). Similar to a previous study by our group (Saavedra et al., 2012), the researcher was in charge of recording the verbalized response of the participants, pressing the left or the right mouse button concomitantly with their verbalization if the response was H or S, respectively, thus recording their response times (RpTs). Two trained researchers recorded the RpTs across all recording sessions without previous knowledge of each participant’s diagnosis.

Half of the participants performed the two direct attention tasks in the global-local order and the other half in the local-global order. Each task was preceded by the corresponding instructions and 12 practice trials. Not until the participant understood and performed the practice trials correctly did the experiment begin. A reminder of the instructions then announced the start of the global or local task as appropriate. On each trial, a hierarchical letter was randomly presented in any of the quadrants of the screen (top left, top right, bottom left, and bottom right) for 500 ms. The peripheral presentation of the hierarchical stimuli and their limited exposure time aimed to avoid inducing local processing facilitation in the participants (cf. section “Introduction”). The inter-trial post-interval, during which the participants had to make their response, was 2000 ms. No feedback was provided during the task. The number of congruent, incongruent, and control trials was equivalent (1/3 trials each, that is, 24 trials per condition except for the last 16 participants who performed 20 trials per condition to minimize fatigue), and their order of appearance was random in each block. Similarly, the presence of the letter H or S was random. To simplify the trial’s structure, a fixation point was not used here, whereas participant’s directed attention was continuously monitored by the researcher that recorded the responses.

#### Data analysis

The mean of raw RpTs collected by the researcher (for correct answers) was analyzed with a linear mixed model with subjects as the random factor, with a fixed between-participant factor and two fixed within-participant factors, establishing a compound symmetry variance-covariance matrix. The independent variables were the between-participant factor *Group* (HE/MCI/AD) and the within-participant factors *Task* (global/local) and stimulus *Congruency* (congruent/incongruent/control).

As the model could not be simplified (by the loglikehood ratio test according to the principle of parsimony), we analyzed the threefold interaction *Group* × *Task* × *Congruency.* Two values were found to have elevated positive skewness of the residual (i.e., abnormally high RpTs), most likely due to distractions at the moment of issuing a response; therefore, residuals were categorized, and extreme atypical cases (which were considered as outliers) were eliminated (–3 < Z_*Residual*_ < 3) following the procedure described by Pardo and San Martín (2015).

Discrimination sensitivity (*d’*) was also calculated. This index, according to the Signal Detection Theory (Green and Swets, 1966), in the present study reveals the ease with which each group of participants could differentiate the letters ‘H’ and ‘S’ in each *Congruency* condition and *Task*. The *d’* was calculated using the formula described in Meyer et al. (1988), where pH denotes the mean probability of generating hits and pFA denotes the mean probability of generating false alarms: $d^{\prime }=0.6\,\text{log}\,e\,\frac{p\,H\,\left(1-p\,F\,A\right)}{p\,F\,A\,\left(1-p\,H\right)}$. Hit and false alarm probabilities with 1 or 0 were adjusted to 0.99 or 0.01, respectively, as per Macmillan and Creelman (2005). The *d’* for each condition of congruency was calculated according to the response categories shown in Table 2.

**TABLE 2:** Response categories for calculating discrimination sensitivity (d’).

	Global			Local	
Congruent	Hits	Correct H_H (response: ‘_H_’)_	Congruent	Hits	Correct H_H (response: ‘_H_’)_
	Fails	Incorrect H_H (response: ‘_S_’)_		Fails	Incorrect H_H (response: ‘_S_’)_
	False Alarms	Incorrect S_S (response: ‘_H_’)_		False Alarms	Incorrect S_S (response: ‘_H_’)_
	Correct Rejections	Correct S_S (response: ‘_S_’)_		Correct Rejections	Correct S_S (response: ‘_S_’)_
Incongruent	Hits	Correct H_S (response: ‘_H_’)_	Incongruent	Hits	Correct S_H (response: ‘H’)_
	Fails	Incorrect H_S (response: ‘_S_’)_		Fails	Incorrect S_H (response: ‘_S_’)_
	False Alarms	Incorrect S_H (response: ‘_H’)		False Alarms	Incorrect H_S (response: ‘_H_’)_
	Correct Rejections	Correct S_H (response: ‘_S_’)_		Correct Rejections	Correct H_S (response: ‘_S_’)_

The hierarchical stimulus corresponding to each response is shown in this table in capital letters for the global letter and in subscript for the local letter. Note that the experimenter arbitrarily decides which stimulus acts as ‘the signal’ and which acts as ‘the noise.’ Thus, we established here that H acted as the ‘signal’ and S as the ‘noise.’ The control condition was not considered for calculating this index.

The *d’* was analyzed by means of a mixed general linear model with subjects as the random factor, with a fixed between-participant factor and two fixed within-participant factors, establishing a compound symmetry variance-covariance matrix. The independent variables were the between-participant factor *Group* (HE/MCI/AD) and the within-participant factors *Task* (global/local) and stimulus *Congruency* (congruent/incongruent). As the model could not be simplified, we selected the most complex model, which comprised the three-fold interaction *Group* × *Task* × *Congruency.*

In both measures (RpTs and *d’*), pairwise comparisons were carried out by the Tukey *post hoc* test, applying the Bonferroni correction for significant effects. Results are presented using Fisher’s *F*-test (95% confidence level), as appropriate for linear mixed models. In accordance with Cohen (1988), effect size was estimated by calculating partial eta-squares (*ηp^2^*).

Speed-accuracy trade-off was measured by correlating the RpTs and the *d’* for each group. The purpose of conducting the analyses was to disentangle whether the costs of better performance could, in this case, be explained by longer visual inspection of the stimulus or, alternatively, whether longer latencies *per se* characterized the responses of our cognitively impaired elderly. Positive correlations would denote a speed-accuracy trade-off, while negative correlations would indicate that the participants take longer to respond in conditions in which they also show poorer discrimination sensitivity.

### Results

#### Response times collected by the researcher

**FIGURE 2:**
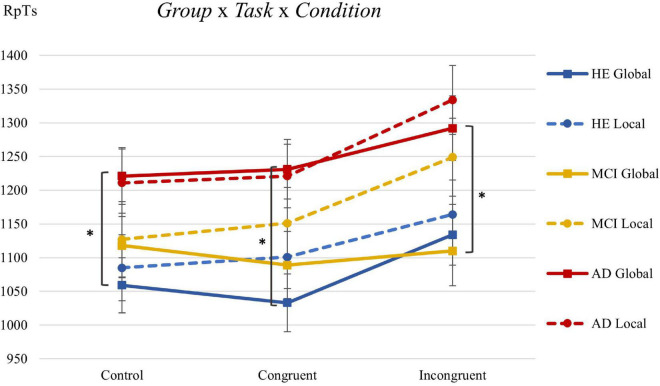
Estimated mean values (and standard error) of RpTs (in ms) for each group, task, and congruency in the Navon task. *Significant differences from pairwise comparisons for the factors *Group* × *Task* × *Congruency*. *Group:* HE, healthy elderly; MCI, mild cognitive impairment; and AD, Alzheimer’s disease. *Task*: global and local. *Congruency*: control, congruent, and incongruent.

Figure 2 shows the mean values of RpTs obtained in the Navon task for each group. The RpTs were clearly slower in both tasks for the AD group than for the HE and MCI groups. Also, the RpTs were, in general, slower in the incongruent condition than in the other two conditions. The statistical analyses showed significant effects for *Group F*(2,399) = 17.2, *p <* 0.001, η*p*^2^ = 0.079 and *Congruency F*(2,272) = 4.63, *p* = 0.011, η*p*^2^ = 0.033: (a) ***Group:*** HE (1096 ms, *p* < 0.001) and MCI (1140 ms, *p* = 0.001) presented faster responses than the AD group (1252 ms), while the hypothesis of absence of differences was maintained for HE and MCI (*p* = 0.38); (b) ***Congruency:*** the congruent (1138 ms, *p* = 0.025) and control (1137 ms, *p* = 0.024) conditions were resolved with faster responses than the incongruent condition (1214 ms), with no differences found between the first two (*p* = 0.99). No differences were found for the *Task* factor *F*(1,398) = 2.90, *p* = 0.089.

Table 3 shows the mean values for RpTs per *Group* × *Task* × *Congruency* (represented graphically in Figure 2), as well as the results derived from the analysis comparing by *Group*. The null hypothesis was maintained (*p* > 0.05) for the absence of differences in the local task. However, in the global task, we found that the HE group was faster than the AD group in the congruent (*p* = 0.006) and control (*p* = 0.022) conditions, and that the MCI group was faster than the AD group in the incongruent condition (*p* = 0.035).

**TABLE 3:** Estimated mean values and statistical results of *Group* × *Task* × *Congruency* comparing mean RpTs and *d’* between *Groups.*

		Global						Local					
Condition	DV	*M*_HE_	*M*_MCI_	*M _*AD*_*	*F* (*df1, df2*)	*p* ^c^	η*p*^2^	*M*_HE_	*M*_MCI_	*M*_*AD*_	*F* (*df1, df2*)	*p^c^*	η*p*^2^
Control	RpTs	1059 ± 41	1118 ± 48	1221 ± 50	3.87 (2, 68)	0.026*	0.10	1085 ± 49	1127 ± 56	1211 ± 50	1.70 (2, 70)	0.19	0.05
Cong.	RpTs	1033 ± 43	1089 ± 50	1231 ± 44	5.46 (2, 68)	0.006*	0.14	1101 ± 47	1151 ± 53	1221 ± 47	1.64 (2, 70)	0.20	0.044
	*d’*	5.09 ± 0.21	5.21 ± 0.24	4.39 ± 0.21	4.18 (2, 68)	0.019*	0.11	5.30 ± 0.15	5.31 ± 0.17	4.68 ± 0.15	5.48 (2, 70)	**0.006** *	0.14
Incong.	RpTs	1134 ± 45	1110 ± 52	1292 ± 48	4.22 (2, 65)	0.019*	0.11	1164 ± 51	1249 ± 58	1334 ± 51	2.80 (2, 70)	0.068	0.07
	*d’*	4.18 ± 0.56	3.76 ± 0.63	2.00 ± 0.56	4.17 (2, 68)	0.020*	0.11	3.62 ± 0.46	3.29 ± 0.51	3.59 ± 0.45	0.14 (2, 70)	0.87	0.004

*M*_HE_, *M*_MCI_, and *M*_AD_: estimated mean values for the healthy elderly, mild cognitive impairment, and Alzheimer’s disease groups, respectively; *M* ± 1.96⋅SE of the mean is shown below of each value. DV, dependent variable; RpTs, response times collected by the researcher; *d*’, discrimination sensitivity. ^c^Bonferroni correction for several comparisons.

*Statistically significant differences (p < 0.05) are highlighted in bold.

It is worth noting that two elderly participants, one of them belonging to the MCI group and the other to the AD group, were unable to identify any of the global figures presented in the trials for training. Thus, no global task was administered to these participants. However, the same participants were able to identify the local elements from the same composed figures; therefore, they did perform the local task. In order to verify whether global interference was affecting these participants despite their failure in overt global form identification, we examined their RpTs in the local task. Thus, we verified that whereas the participant with AD performed the local task in the presence of global distractors at no cost (1378 ms incongruent–1414 ms congruent = –36 ms), the participant with MCI did reveal a notable cost for the presence of global distractors in this task (1780 ms incongruent–1386 ms congruent = 394 ms).

#### Analyses of discrimination sensitivity

**FIGURE 3:**
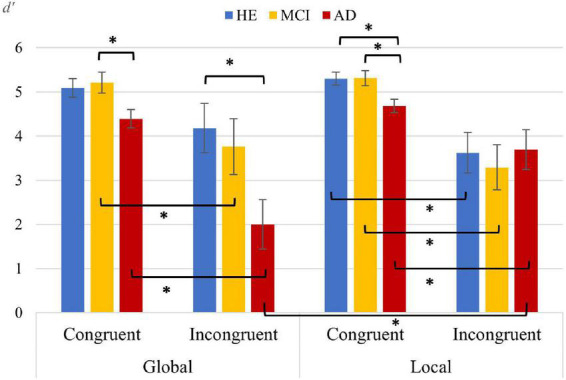
Estimated mean values (and standard error) of *d’* for each group, task, and congruency in the Navon task. *Significant differences from pairwise comparisons for the factors *Group* × *Task* × *Congruency. Group:* HE, healthy elderly; MCI, mild cognitive impairment; and AD, Alzheimer’s disease. *Task*: global and local. *Congruency*: congruent and incongruent.

Supplementary_Material_Studies 1 and 2_discrimination sensitivity (Supplementary Table 2.2.a) shows hit and false alarm probabilities for each group in the different experimental conditions. In general, the *d’* values indicated that the task was relatively easy to perform. Many items were easily discriminable, and the error probability was very low. Thus, the resulting *d’* was very high, mainly in the HE group. Figure 3 illustrates that the AD group reflected worse discrimination sensitivity than the other groups, except in the incongruent condition of the local task. In addition, in this same task, all the groups exhibited better discrimination sensitivity in the congruent condition than in the incongruent condition. Apparently, there are no visible differences in *d’* between tasks. The statistical analyses showed that the main effect *Task F*(1,164) = 0.71, *p* = 0.4 was not significant. Conversely, the main effects *Group F*(2,164) = 5.93, *p* = 0.003, η*p*^2^ = 0.067 and *Congruency F*(1,164) = 47.6, *p* < 0.001, η*p*^2^ = 0.22 were found to be significant. In particular, (a) ***Group****:* HE (4.55, *p* = 0.004) and MCI (4.39, *p* = 0.038) presented better discrimination than the AD group (3.67) and, (b) ***Congruency:*** the congruent (5, *p* < 0.001) condition was discriminated better than the incongruent condition (3.41). Relevantly, the analysis of the interaction *Group* × *Task* × *Congruency* did reveal differences comparing by *Task.* In particular, in the incongruent condition, AD showed better discrimination of the letters *F*(1,131) = 4.87, *p* = 0.029, η*p*^2^ = 0.036 in the local than in the global task. The same interaction comparing by *Congruency* showed significant differences between conditions, revealing better discrimination in the congruent than the incongruent condition in the MCI [*F*(1,87) = 4.59, *p* = 0.035, η*p*^2^ = 0.05) and AD [*F*(1,87) = 15.8, *p* < 0.001, η*p*^2^ = 0.15] groups in the global task and in all the groups in the local task [HE: *F*(1,85) = 12.4, *p* = 0.001, η*p*^2^ = 0.13; MCI: *F*(1,85) = 14.3, *p* < 0.001, η*p*^2^ = 0.14; AD: *F*(1,85) = 5.31, *p* = 0.024, η*p*^2^=0.02]. No differences were found between conditions for the HE group in the global task (*p* > 0.05).

Table 3 shows pairwise comparisons among *Groups* in *d’*, considering *Task* and *Congruency*, represented graphically in Figure 3. The pairwise comparisons revealed significantly better discrimination of the letters in HE than in AD in the incongruent condition of the global task (*p* = 0.023) and in the congruent condition of the local task (*p* = 0.015). Also, the MCI group revealed better discrimination of the letters than AD in the congruent condition of both global (*p* = 0.035) and local (*p* = 0.022) tasks.

#### Speed-accuracy trade-off

Regarding the speed-accuracy trade-off analysis, we found significant negative correlations (i.e., slower responses were associated to poorer discrimination, thus revealing the absence of speed-accuracy trade-off) in the congruent condition of the local task in both the MCI (*r* = –0.49, *p* = 0.025) and AD (*r = –*0.54, *p* = 0.004) groups. No significant correlations were observed in the rest of the comparisons.

### Discussion

#### Severity of cognitive impairment modulated the global/local visual processing

The main aim of this study was to test whether the GPE that defines visual perception of hierarchical stimuli in young adults, characterized by a global advantage and unidirectional global interference, changes during typical aging and even more so in elderly people with MCI and AD. Our results revealed, as a common finding in the performance in the Navon task among the different groups of participants, slower RpTs in the incongruent vs. the congruent condition irrespective of the task performed, reflecting a bidirectional interference effect. Moreover, the RpTs showed no differences between the global and local tasks in the congruent condition, reflecting a lack of global advantage and thus providing evidence for attenuation of the GPE in elderly people.

In addition, we noted a common absence of speed-accuracy trade-off, which was even revealed as negative correlations in the congruent condition of the local task in both MCI and AD. This differs from the effect that has characterized the performance of young adults who displayed both faster reaction times and better performance in a similar task (Álvarez-San Millán et al., 2021, 2022).

In the next paragraphs, we will discuss the results observed as more specific profiles in each group of older adults.

##### No global advantage and some unidirectional global interference were observed in the healthy elderly group

A singular finding in the HE group was the evidence of some unidirectional global interference (i.e., global distractors hampered local task performance, whereas local distractors did not interfere in the global task) when discrimination sensitivity was analyzed. However, like in the other groups, no global advantage (i.e., no faster responses were observed in the congruent global vs. local task) was observed, suggesting a decrease in the GPE in this group of participants. These results contradict the local facilitation described in healthy older adults by Oken et al. (1999), Slavin et al. (2002), and Lux et al. (2008), whereas they partially support the absence of differences between the global and local identifications reported by Filoteo et al. (1992, 1995) and Staudinger et al. (2011). Also, the results obtained are partially comparable to those reported by authors who observed, in addition to a global advantage, a unidirectional global interference, i.e., an interference of the irrelevant level during local processing but not during global processing (Bruyer and Scailquin, 2000; Roux and Ceccaldi, 2001; Bruyer et al., 2003).

##### The mild cognitive impairment group showed no global advantage and bidirectional interference with a bias to process the irrelevant local letter

The elders with MCI also showed no global advantage and, unlike HE, a clear bidirectional interference (i.e., slower RpTs in incongruent vs. congruent trials in both tasks) besides a decreased discrimination sensitivity in the incongruent condition of both the global and local tasks, thus denoting higher vulnerability than HE to the presence of distractors in general. The inability to inhibit attention to the irrelevant level distractor independently of the attended level could reflect an age-related decline of the capacity to inhibit distractors during task-specific focus according to the inhibition-deficit theory of aging (Hasher et al., 2007). This idea was already supported by Tsvetanov et al. (2013) who, manipulating hierarchical letter salience, found that older participants were more affected than younger participants by salient distractors at the irrelevant level, suggesting the existence of a general inhibitory control deficit. Also, Wiegand et al. (2015) found severe impairment in older adults to be inhibiting global distractors during local processing in a study on ERPs in which Kanizsa figures were used as stimuli. With regard to neural bases, the inhibitory control necessary for the correct performance of incongruent trials has been related to the predominantly right activation of the middle frontal gyrus, superior frontal gyrus, and inferior parietal lobe (Hedden and Gabrieli, 2010), so the more rapid and marked atrophy of the right hemisphere during aging (Dolcos et al., 2002) could also explain to some extent the decline in inhibitory processes observed in the present experiment.

Our results support our hypothesis that in MCI we would find greater attenuation of GPE when contrasted with HE, revealing an intermediate evolutionary moment between healthy aging and dementia, as has been found in other visual processing tasks (Spoletini et al., 2008). The previous literature has not addressed differences between HE and MCI in global/local visual processing, so the present study is the first to reveal a clear attenuation of the GPE in premorbid stages of AD in this type of task. Moreover, the speed-accuracy trade-off analysis revealed a negative correlation between the RpTs and *d’* in the congruent trials of the local task, which could reflect that far from benefiting from the facilitation of the same letter being at both levels of the hierarchical stimulus, participants in the MCI group spent a more unproductive time in responding to the trials.

It should also be recalled that the MCI group had the highest percentage of participants (28.6%) under treatment with benzodiazepines. However, although the use of these drugs in young people has been associated with problems in attention, visuospatial processing, and processing speed (Barker et al., 2004), their effects on the neuropsychological test performance of octogenarian participants have only been associated with deficits in oral comprehension and verbal memory (Helmes and Østbye, 2015).

##### Participants with Alzheimer’s disease showed no global advantage, bidirectional interference, and enhanced bias to the irrelevant local letter with the slowest responses and the worst discrimination sensitivity

A particular feature associated to the profile of AD in the present study was the generalized slower responses in the participants when contrasted with the HE and MCI participants (main factor *Group* was significant when RpTs were analyzed), and more specifically, the significantly slower responses in the global task of this group than the other groups (HE faster than AD in control and congruent conditions; MCI faster than AD in incongruent condition). Additionally, in the elderly people with AD, we did not find the local advantage reported in previous studies (Coslett et al., 1995; Slavin et al., 2002; Blanca et al., 2010), but like Filoteo et al. (1992, 1995), in these patients we observed no difference between global and local RpTs corresponding to the congruent condition. In this group, we also observed bidirectional interference, that is, slower responses in the incongruent vs. the congruent condition in both tasks.

As for *d’*, this group’s overall performance was poorer than MCI and HE (main factor *Group* was significant), with the peculiarity of better discrimination of letters in the local than in the global task in the incongruent condition in AD, thus denoting lower sensitivity to the presence of global distractors. It is worth noting that both MCI and AD, in contrast to HE, displayed better discrimination in the congruent condition than in the incongruent condition in the global task, suggesting that the irrelevant local level was highly disruptive in the two groups. Similarly, the speed-accuracy trade-off analysis revealed that this group, like the MCI group, spent more time and made a greater number of errors in the congruent condition of the local task, which could reflect an inefficient slowdown associated to the least demanding condition in both groups.

It should also be noted that in this experiment one participant with MCI and one with AD were not able to identify the global level of the composite figures despite repeated practice. The inability to form the global percept in some patients with AD has already been reported by other authors (Coslett et al., 1995; Slavin et al., 2002; Piccini et al., 2003), which could be explained as severe impairment of the process of grouping the elements that make up a visual scene, a function that we indicated in the Introduction section to be impaired in healthy older adults (Staudinger et al., 2011; Lithfous et al., 2016) and in those with AD (Kurylo et al., 2003). Relevantly, Slavin et al. (2002) have pointed out that such participants, despite being unable to perceive the global figure, do show global interference during local task processing, revealing implicit processing of the global shape. This same finding was obtained with our participant with MCI, who revealed a possible global covert interference, but not in our patient with AD, who showed slightly shorter RpTs in the incongruent condition than in the congruent condition.

## Study 2: One-year follow-up of global/local visual processing in healthy elderly, mild cognitive impairment, and Alzheimer’s disease, and analysis of its relationship with performance in neuropsychological tests sensitive to cognitive impairment

The results found in the previous cross-sectional study led us to conduct a longitudinal study. The Navon letter task was repeated 1 year later to the same available participants. Our purpose was to test whether global/local visual processing undergoes a differential deterioration in each studied group over time and whether the results obtained in the Navon task in the 2nd moment of administration can be related to dysfunction in certain cognitive domains as neurodegeneration progresses. We expected to find no differences in HEs given that they did not suffer from any degenerative pathology that could significantly alter their global/local visual processing in the short or medium term. Regarding the adults with MCI, we expected to observe a more accentuated impairment of global processing in participants whose diagnosis had worsened, remaining unchanged in MCI participants who maintained their initial diagnosis. Finally, we expected that the participants with AD, with more pronounced exposure to neurodegenerative factors and the worst performance in the first study, would be even more affected in their global visual processing after 1 year, in line with the results observed in previous cross-sectional studies (Slavin et al., 2002; Blanca et al., 2010). Furthermore, given that in the discussion of the cross-sectional study we suggested the existence of a possible inability to inhibit the irrelevant local level in the global task in all the groups, as well as a least sensitivity of AD to the irrelevant global stimuli in the incongruent trials of the local task, we expected to observe an accentuation of over time differences in the incongruent condition.

Additionally, we examined which cognitive domains could be more performance-related in the global/local visual processing task and, specifically, whether a deficit in inhibitory control could contribute to explain the results observed in the incongruent condition in the cross-sectional study. To this end, we carried out an exploratory study in which a neuropsychological battery with high sensitivity and specificity to cognitive impairment in elderly people was administered to participants in the 2nd moment of application of the Navon letter task. We hypothesized that the scores obtained by our participants in neuropsychological tests on executive functions should be related to the RpTs obtained in the global and local tasks. In particular, we expected that certain indexes of the neuropsychological battery related to executive functions would allow us to differentiate, together with the results of the Navon letter task, between healthy older adults and those with cognitive impairment, as well as to advance in the identification of early cognitive markers of the onset of dementia of Alzheimer’s type.

### Methods

#### Participants

Approximately 1 year after the first administration (*M* = 361 days; *SD* = 80.7), the participants were followed up in the Navon letter task, applied under the same conditions to those who were available. Due to the characteristics of the studied population, there was a high experimental mortality [59.1% (26 participants) due to voluntary or residential dropout, 15.9% (seven participants) due to death, 18.2% (eight participants) due to disabling illness, and 6.8% (three participants) due to severe worsening of cognitive impairment. In addition, according to the NIA-AA criteria (Mckhann et al., 2011; Jack et al., 2018), four of the participants diagnosed with MCI were also diagnosed with probable AD after 1 year. Accordingly, we differentiated in the longitudinal study between older participants with a diagnosis of MCI at both assessment moments (MCI) and participants with MCI who at the 2nd assessment moment had a diagnosis of probable AD (MCI/AD). The remaining elderly participants had no change in their diagnosis. Altogether, 29 participants were followed up, including 9 HE, 5 MCI, 4 MCI/AD, and 11 AD. Table 4 describes the sample characteristics of each group of elderly participants at the 2nd moment of assessment.

**TABLE 4:** Sample characteristics of the longitudinal study with four groups of elderly participants, incorporating a group whose initial diagnosis of MCI was changed to AD at the 2nd moment of assessment (MCI/AD group).

	HE (*n* = 9)	MCI (*n* = 5)	MCI/AD (*n* = 4)	AD (*n* = 11)	χ^2^ (*df*)	*p*(η*p*^2^)
Age	88.5 (4.10)	87.6 (7.92)	90.3 (6.94)	84.7 (6.13)	3.38 (3)	0.34
Sex (M:F)	6 : 3	3 : 2	0 : 4	3 : 8	6.58 (3)	0.087
Education (I/II/III/IV)	(0/6/1/2)	(0/5/0/0)	(0/3/0/1)	(1/7/2/1)	1.75 (3)	0.63
Handedness	1.11 (0.17)	1.24 (0.23)	1.10 (0.17)	1.27 (0.58)	1.38 (3)	0.71
MMSE	28.3 (1.5)	26.0 (1.67)	21.8 (2.59)	16.4 (5.61)	22.2 (3)	<0.001* (0.46)
GDS	1.11 (0.31)	2.00 (0)	3.00 (0)	3.82 (0.83)	24.9 (3)	<0.001* (0.49)

Sex: M, male and F, female. Level of education: I, no schooling; II, elementary school; III, secondary school; and IV, graduate school. MMSE, Spanish version of the Mini-Mental Status Examination; GDS, Global Deterioration Scale; HE, healthy elderly; MCI, mild cognitive impairment; MCI/AD, participants with MCI who progressed to probable AD; AD, Alzheimer’s disease.

*Statistically significant differences (*p* < 0.05) are highlighted in bold.

By means of the Kruskal–Wallis test, it was not possible to affirm the existence of differences between the groups in the variables age, sex, educational level, and manual laterality, but it was possible to affirm the existence of differences in the MMSE and GDS scores. In particular: (a) MMSE: it was found that participants in the AD and MCI/AD groups had a lower score than those in the HE group, and that those in the AD group had a lower score than those in the MCI group; (b) GDS: it was observed that participants in the AD, MCI/AD, and MCI groups had a higher score on the scale than those in the HE group, and those in the AD and MCI/AD groups had a higher score than those in the MCI group. On the other hand, we examined whether the participants had experienced worsening of their cognitive and functional functioning between the 1st and 2nd moments of the assessment. By Wilcoxon signed-rank test, the null hypothesis that MMSE scores did not differ between the 1st and 2nd moments of the assessment in the HE (*z* = –1.52, *p* = 0.13), MCI (*z* = –1.34, *p* = 0.18), and MCI/AD (*z* = –1.6, *p* = 0.11) groups was maintained, while worsening of cognitive impairment was found in the AD group (*z* = –2.24, *p* = 0.025, η*p*^2^ = 0.45). Regarding GDS score, the Wilcoxon signed-rank test revealed that GDS scores did not differ between the 1st and 2nd moments of the assessment in either group (*p* > 0.05). Note that the small sample size in MCI/AD (four participants) might explain the lack of significant differences in MMSE score from the MCI group. Nonetheless, the average score was 26 in MCI and 21.8 in MCI/AD, denoting a drop of more than 4 points. Moreover, we did reach to observe significant differences in the GDS between the two groups (GDSs 2 and 3 respectively).

#### Materials and procedure

The materials and procedure used in the Navon letter task were the same as those applied in study 1, with 60 trials for each global and local task being presented to all the participants on this occasion. Additionally, in study 2 a neuropsychological battery, with both high sensitivity and specificity to cognitive impairment in elderly people, was administered which, due to its length, was divided into four sessions of approximately 30 min each. The battery included verbal memory tasks (logical memory, subtest WMS-IV, Wechsler, 2013; word list, subtest WMS-III, Wechsler, 2004), visual memory (delayed matching-to-sample task 48, Barbeau et al., 2004), language (Boston naming test 12-item version, Serrano et al., 2001; categorical verbal fluency test: animals and names of people, Saez-Zea et al., 2008), and executive functions (phonemic verbal fluency task: letters ‘A’ and ‘S,’ Goñí-Sarriés et al., 2015; automatic/inhibitory control subtest: item of the BSCE-WMS-IV screening test, Wechsler, 2013; Hayling test: automatic/inhibitory part, Burgess and Shallice, 1997, Spanish adapted version, Pérez-Pérez et al., 2016; digit span test: forward and backward, WAIS-IV, Wechsler, 2012; trail-making test black and white version: TMT B&W A and B, Kim et al., 2014). Group differences in neuropsychological assessment can be found in Supplementary_Material_Study 2_Neuropsychological assessment (Supplementary Table 4.2.a.). It should be noted that the only neuropsychological index that revealed a differential performance between the MCI and MCI/AD groups was the number of correct scores on the Boston naming test, with a higher score in the MCI group than in the MCI/AD group.

#### Data analysis

A very high experimental mortality 1 year on from the 1st task administration caused a notable reduction in the number of participants who performed the Navon task in the 2nd moment of the assessment. Consequently, we carried out one-way ANOVAs using the factor *Group* and pairwise comparisons of those RpTs obtained in the Navon task at the 2nd moment of the assessment. We also used *Change over time index* [as described below in point “Change over time index (I_1–2_)”] to analyze the changes in the Navon task between the first and the second applications, as well as in order to reduce the number of parameters to be analyzed. Moreover, we carried out an assessment of the individual change over time in RpTs (as described below in point “Assessment of the individual change over time in RpTs”). Lastly, the measures derived from the one-way ANOVAs were then correlated with those obtained from the neuropsychological battery administered in the present study.

##### Navon task 1 year later

Using the RpTs from correct responses obtained at the 2nd moment of the assessment, we tested differences between groups carrying out a one-way ANOVA for each dependent variable of the Navon task (global congruent, global incongruent, global control, local congruent, local incongruent, and local control) and applying the Brown–Forsythe adjustment of degrees of freedom and Games–Howell *post hoc* pairwise comparison test when the hypothesis of homogeneity variances was not fulfilled.

##### Change over time index (I_1–2_)

*Change over time index* (I_1–2_) was obtained by subtracting the RpTs/*d’* registered in the 2nd moment of the assessment from the RpTs/*d’* registered in the 1st moment (that is, 1st moment *minus* 2nd moment) for every participant and independently for each task. This allowed us to verify whether the change 1 year later was similar or not in both tasks. A negative value in this index would reflect a slowdown in the 2nd vs. the 1st moment in the task, whereas a positive value would reflect an acceleration of the RpTs. As for *d’*, a negative value would reflect better discrimination in the 2nd vs. the 1st moment in the task, whereas a positive value would reflect worse performance within each task. This index is a measure of performance over time independently for each task. Thus, the existence of differences between both tasks would suggest that one task might be significantly more affected 1 year later.

We analyzed the *Change over time index (I_1–2_)* in the different groups for every task and condition of congruence. The independent variables were the between-participants factor *Group* (HE/MCI/MCI-AD/AD) and the within-participant factors *Task* (global/local) and *Congruency* (congruent/incongruent/control). We carried out an approximation for small sample sizes for the linear mixed models with repeated measures, particularly the Satterthwait degrees of freedom approximation that control type I errors (Huber et al., 2015), with a compound symmetry variance-covariance matrix, with random intercepts (participants) and slope (assessment moment).

##### Assessment of the individual change over time in response times collected by the researcher

Additionally, considering both the reduced size of our sample and possible differences among the participants within each group, we carried out an individualized analysis of the RpTs, since it would be very informative to study a possible change in this variable between the 1st and 2nd moments of the assessment in the different measures derived from the global/local task. Thus, we selected the statistic *index of efficiency* (i.e., individual typified difference) as an index of individual changes that offers the best results when a criterion of ± 1.96 is applied in the statistic (Pardo and Ferrer, 2013). This approach consists of typifying every pre-post difference by dividing it by its standard deviation (i.e., the standard deviation of the differences), considering significant values above 1.96 or below –1.96.

##### Neuropsychological variables analyzed in relation to global/local visual processing

In order to explore whether performance in the Navon task might be related with performance in neuropsychological tests with both high sensitivity and specificity to cognitive impairment in elderly people, a correlation analysis was carried out considering 25 measures derived from the neuropsychological assessment and 6 ‘perceptual’ measures from the Navon task. The variables included scores obtained in the neuropsychological battery from several domains such as verbal memory (logical memory; word List), visual memory (DMS48), language (Boston naming test; categorical fluency verbal test), and executive functions (phonemic fluency verbal test; automatic/inhibitory control subtest; Hayling test; digit span test; TMT B&W). Since a Gaussian distribution could not be assumed (by the Shapiro–Wilk test) for 18 of the 31 variables, Spearman’s correlation was calculated for all the variables (the sample size ranged from 18 to 29 cases after elimination of some outliers in some variables).

### Results

#### Navon task 1 year later

Table 5 shows the results derived from the one-way ANOVAs and pair comparisons of RpTs in each group of older adults for each condition of the Navon task at the 2nd moment of the assessment. The analysis revealed faster responses in the HE group than in the AD group in both congruent and control conditions of the global task and in the control condition of the local task.

**TABLE 5:** Results of one-way ANOVAs using the factor *Group* and pairwise comparisons of the RpTs obtained in the Navon task at the 2nd moment of the assessment.

Dependent variable	HE	MCI	MCI/AD	AD	*p*(η*p*^2^)	Bonferroni
Global control^a^	923 ± 54.5	1221 ± 88.6	1017 ± 13.5	1178 ± 66.7	0.009* (0.49)	**HE < AD**
Global congruent^a^	936 ± 49.6	1137 ± 90.1	1071 ± 14.7	1204 ± 72.8	0.020* (0.44)	**HE < AD**
Global incongruent	1014 ± 48.8	1055 ± 107	1040 ± 32.2	1237 ± 66.5	0.077	–
Local control	945 ± 40.4	1218 ± 94.3	1099 ± 99.0	1179 ± 60.1	0.026* (0.30)	**HE < AD**
Local congruent	972 ± 49.5	1307 ± 107	1157 ± 102	1200 ± 74.7	0.041	n.s.
Local incongruent	1054 ± 47.3	1353 ± 156	1315 ± 171	1343 ± 96.7	0.126	–

HE, healthy elderly; MCI, mild cognitive impairment; MCI/AD, participants with MCI who progressed to probable AD; AD, Alzheimer’s disease.

^a^Levene’s test indicated unequal variances, so degrees of freedom were adjusted by Brown–Forsythe, and a Games–Howell *post hoc* pairwise comparison test was performed.

*Statistically significant differences (*p* < 0.05), highlighted in bold. ‘ < ’ means faster RpTs.

#### Change over time index (I_1–2_)

**FIGURE 4:**
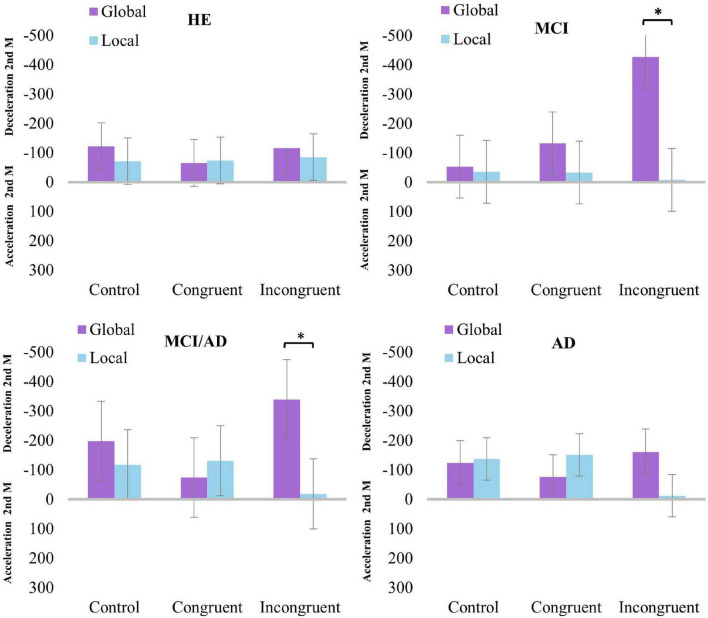
Results of pairwise comparisons for *Group* × *Congruency* × *Task* comparing the I_1–2_RpTs index between the global and local tasks. *Statistically significant differences (*p* < 0.05). Error bars show the standard error of the mean. Note that for the sake of clarity, negative values in the *Y*-axis are represented pointing up.

Supplementary Table 5.2.a of Supplementary_ Material_Study 2_Response times shows the estimated I_1–2_RpTs mean values as well as derived results from pairwise comparisons as per the factor *Task*. Significant or marginally significant differences are represented graphically in Figure 4. Based on the analyses, we could not support the existence of significant differences in I_1–2_ in both congruent and control conditions. In turn, the results revealed that in the incongruent condition, larger slowdown was found in the 2nd moment of the assessment in the global vs. local task in both the MCI and MCI/AD groups.

Additionally, the analysis of index I_1–2_*d’* from pair comparisons as per the factor *Task* revealed that the AD group in the incongruent condition showed better discrimination of local than global letters in the 2nd moment of the assessment *F*(1,72) = 5.97, *p* = 0.017, ηp^2^ = 0.076. In the congruent and control conditions, we could not sustain the existence of significant differences in the index I_1–2_ *d*’. The averaged (and SD) hit and false alarm (FA) probabilities for each group in the different experimental conditions as well as the estimated mean values and results of pairwise comparisons for *Group* × *Congruency* × *Task* comparing the I_1–2_*d’* between the global and local tasks can be found in Supplementary_Material_Studies 1 and 2_Discrimination sensitivity (Supplementary Tables 5.2.b, 5.2.c).

#### Individual change in participants over time

Supplementary Table 5.3.a of Supplementary_ Material_Study 2_Response times shows the number of participants in each group who presented a significant change in performance after 1 year for each task and condition. The analysis of the relevant individual change discarded the existence of differences between the 1st and 2nd moments of the assessment for all participants in the HE group. As for the MCI group, two participants revealed slowdown in performance in the Navon task in the re-evaluation, one of them in the incongruent condition of the global task (1st: 607 ms vs. 2nd: 1902 ms) and the other in the incongruent condition of the local task (1st: 1357 ms vs. 2nd: 1901 ms). Nonetheless, a third participant in the MCI group showed acceleration in the 2nd moment of the assessment in the incongruent condition of the local task (1st: 1882 ms vs. 2nd: 1215 ms). With regard to the MCI/AD group, only one out of four participants showed a relevant change after 1 year, that is, slowdown in the congruent (1st: 1100 ms vs. 2nd: 1544 ms), incongruent (1st: 1103 ms vs. 2nd: 2004 ms), and control (1st: 1036 ms vs. 2nd: 1657 ms) conditions of the global task as well as in the control condition (1st: 812 ms vs. 2nd: 1144 ms) of the local task. Finally, five participants in the AD group showed significant slowdown in their RpTs in the 2nd moment of the assessment. This occurred with two participants exclusively in the control condition of the local task (1st: 889 ms vs. 2nd: 1225 ms; 1st: 1401 ms vs. 2nd: 1759 ms), whereas the other three participants displayed slowdown in at least one condition of both the global and local tasks.

#### Exploratory neuropsychological study in relation to global/local visual processing

The Spearman correlations between neuropsychological variables and the different conditions of the Navon task can be found in Table 6. The analysis revealed that all the ‘perceptual’ (Navon) variables are highly correlated with several neuropsychological variables, mainly relating to both language and executive functions, although a few of them with memory, mainly with visual memory. This is especially noticeable in the congruent condition of the global task.

**TABLE 6:** Results of Spearman correlations between neuropsychological variables and the different conditions of the Navon task.

Cognitive domain		Perception – Navon task (RpTs)					
	Neuropsychological test	Global control	Global congruent	Global incongruent	Local control	Local congruent	Local incongruent
Memory	LM I: immediate (hits)	–0.48	**–0**.**60**	–0.37	–0.46	–0.44	–0.44
	LM II: delayed (hits)	–0.37	–0.47	–0.40	–0.26	–0.30	–0.31
	LM II: recognition (hits)	–0.45	**–0**.**57**	–0.36	–0.34	–0.33	–0.36
	WL I: immediate (hits)	–0.43	–0.47	–0.23	–0.40	–0.28	–0.37
	WL II: delayed (hits)	–0.14	–0.32	–0.19	–0.40	–0.34	–0.38
	WL II: recognition (hits)	–0.22	–0.34	–0.28	–0.03	0.00	0.00
	DMS: Set1 Unique (error)	0.12	0.23	0.44	0.31	0.26	0.19
	DMS: Set1 Double (error)	0.16	0.31	0.43	0.11	0.12	0.12
	DMS: Set1 Abstract (error)	0.40	0.42	0.37	0.32	0.33	0.22
	DMS: Set2 Unique (error)	0.35	0.45	0.47	**0.52**	0.46	0.42
	DMS: Set2 Double (error)	0.26	0.20	0.18	0.37	0.38	0.23
	DMS: Set2 Abstract (error)	0.50	**0.54**	0.38	**0.57**	**0.54**	0.48
Language	Animal fluency (hits)	**–0**.**59**	**–0**.**76**	**–0**.**56**	**–0**.**53**	–0.49	**–0**.**55**
	Names fluency (hits)	**–0**.**58**	**–0**.**72**	–0.48	**–0**.**53**	–0.48	**–0**.**57**
	Boston naming test (hits)	–0.45	**–0**.**59**	–0.41	–0.27	–0.22	–0.37
Executive functions	‘S’ fluency (hits)	**–0**.**58**	**–0**.**76**	–0.46	**–0**.**62**	**–0**.**53**	**–0**.**67**
	‘A’ fluency (hits)	–0.46	**–0**.**64**	–0.34	–0.41	–0.34	–0.39
	Auto. Control latency (RT)	**0.64**	**0.81**	**0.63**	0.49	0.42	**0.51**
	Inhib. Control (error)	0.48	**0.57**	**0.65**	0.35	0.32	0.35
	Auto. Hayling latency (RT)	0.22	0.32	0.32	0.26	0.20	0.22
	Inhib. Hayling (error)	0.43	**0.55**	0.42	0.40	0.36	0.39
	Digit span Forward (hits)	**–0**.**55**	**–0**.**66**	–0.25	**–0**.**71**	**–0**.**60**	**–0**.**68**
	Digit span Backward (hits)	**–0**.**66**	**–0**.**61**	–0.28	–0.42	–0.35	–0.36
	TMT B&W – A (RT)	**0.61**	**0.71**	**0.53**	0.39	0.41	**0.59**
	TMT B&W – B (RT)	**0.60**	**0.62**	**0.60**	0.33	0.30	0.48

LM, logical memory; WL, word list; DMS, delayed matching-to-sample task 48; TMT B&W, trail-making test black and white version; and RpTs, response times collected by the researcher. Bold and underlined values represent correlations greater than 0.5.

### Discussion

#### Global visual processing declines more markedly after 1 year in elderly people with cognitive impairment

To our knowledge, this is the first longitudinal study assessing global/local visual processing using a Navon letter perception task in elderly people 1 year after the 1st administration. To test the evolution in behavioral performance, we analyzed *Change over time index* (I_1–2_) as well as the individual change of each participant using the Individual Typified Difference method. Additionally, performance on the global/local visual processing of hierarchical stimuli was analyzed in relation to performance on a series of neuropsychological tests sensitive to cognitive impairment. The results revealed that at the 2nd moment of the assessment: (a) RpTs were significantly faster in the HE group than in the AD group in several conditions, and that no differences were detected among the rest of the groups; (b) none of the participants belonging to the HE group revealed a significant individual change between the 1st and 2nd moments of the assessment; (c) there was greater slowing of global vs. local processing in the MCI and MCI/AD groups and marginally in the AD group; (d) the AD group showed better discrimination of local than global letters in the incongruent condition; (e) perceptual variables of the Navon task were correlated with several neuropsychological variables related to language, executive functions and (visual) memory.

It is worth highlighting first that the differences found in the longitudinal study in *Change over time index* (*I****_1–2_***) were found in the incongruent condition. We interpret that this condition is the most sensitive to cognitive changes over time, probably because it is the one that requires more cognitive demands as it involves inhibitory processes that are susceptible to age-related decline, as pointed out by Hasher et al. (2007). Thus, in the MCI, MCI/AD, and AD groups (in the third case as revealed by discrimination sensitivity), the locally irrelevant letter interfered with the processing of the global target letter more markedly in the 2nd moment of the assessment than in the 1st moment. Although we are not aware of previous longitudinal studies, the results are consistent with those found by other authors in cross-sectional studies, which underline a more rapid or prioritized analysis of local vs. global elements in older participants with cognitive impairment (Coslett et al., 1995; Slavin et al., 2002; Blanca et al., 2010). As expected, the HE group, whose diagnosis did not change after 1 year, did not show worsening of their cognitive functions and maintained similar global and local visual processing at both times of task administration. It is worth highlighting that unlike the RpTs with this index, which showed only marginally significant differences in the AD group, the analysis with *d’* did show significant differences between the two administration times in this group. This highlights the relevance of analyzing both variables, as they provide complementary information on changes in global/local visual processing with progression of cognitive impairment.

With regard to relevant individual change, it is worth highlighting that no participant in the HE group revealed a change in global/local visual processing after 1 year, and that in the MCI, MCI/AD, and AD groups at least one participant revealed worse performance in some tasks and conditions over time. These results reflect that passage of time is not a sufficient explanation for the differences found in global/local visual processing in the groups with cognitive impairment. In this regard, it should be mentioned that the only participant who revealed an improvement in his performance in the 2nd moment of the assessment belonged to the MCI group and that it was in the incongruent condition of the local task, which could reflect that after 1 year this participant more easily ignored or inhibited the global irrelevant level distractor during the execution of the local task.

#### Neuropsychological variables related to performance in the Navon task

Regarding neuropsychological variables and their correlation with RpTs in the Navon task, our results suggest that performance in this task may have a diagnostic value similar to the performance assessed by neuropsychological tests, which have proved to be highly sensitive to cognitive aging. However, it is worth noting that this is an exploratory study, and we cannot rule out that the small sample size prevents us from observing more defined cognitive profiles related to global/local processing. In fact, people with MCI have been characterized by their neuropsychological heterogeneity (Petersen, 2004). A larger sample of elderly people might establish a clearer view of a possible differentiation in profiles of cognitive decline correlated with global/local perception. In any event, the analysis of the possible relationship among neuropsychological variables, global/local processing, and cognitive decline, also suggested by other studies (Delis et al., 1992; Massman et al., 1993; Aziz et al., 2021), should include neuropsychological assessment in different moments of a longitudinal study. Furthermore, the application of regression analyses to investigate if Navon measures, complementarily to neuropsychological variables, are valid predictors of progressive cognitive decline, might offer important insights into the nature of different profiles of cognitive impairment associated to AD.

### Summary of results of both cross-sectional and longitudinal studies

Table 7 provides a summary of the main results concerning RpTs and *d’* found in both cross-sectional and longitudinal studies in order to facilitate the integration of our findings. In the cross-sectional study, we identified the significant and non-significant results derived from the comparison of the different conditions (i.e., control, congruent, and incongruent), as well as from speed-accuracy trade-off analyses for each task. Note that both differences and similarities among the HE, MCI, and AD groups are fully revealed when, besides RpTs, discrimination sensitivity and speed-accuracy trade-off are considered. As for the longitudinal study, the enhanced relevance of a bias toward local processing is also clearly revealed when *Change over time index* (I**_1–2_**) is analyzed in both RpTs and *d’*, whereas the results concerning *Individual change in participants over time* are in line with the two measures. Lastly, we summarized the correlations greater than 0.5 of performance in the Navon task per task and congruency with several neuropsychological variables.

**TABLE 7:** Summary of the main findings found in both the cross-sectional and longitudinal studies.

		**Global visual task^a^**			**Local visual task**		
	**Measure**	**Control**	**Congruent**	**Incongruent**	**Control**	**Congruent**	**Incongruent**
	
	RpTs	HE < AD	HE < AD	MCI < AD	n.s.	n.s.	n.s.
Cross-sectional study	*d’*	–	MCI > AD	HE > AD	–	HE > AD MCI > AD	n.s.
	Sp-Acc Trade-off	–	n.s.	n.s.	–	n.s.^b^	n.s.

		**Global visual task**			**Local visual task**		
	**Measure**	**Control**	**Congruent**	**Incongruent**	**Control**	**Congruent**	**Incongruent**
	
	RpTs	HE < AD	HE < AD	n.s.	HE < AD	n.s.	n.s.
	
		**Better in global than in local task at the 2nd moment**			**Better in local than in global task at the 2nd moment**		
	**Measure**	**Control**	**Congruent**	**Incongruent**	**Control**	**Congruent**	**Incongruent**
	
**Longitudinal study**	I_1–2_RpTs	n.s.	n.s.	n.s.	n.s.	n.s.	MCI MCI/AD
	I_1–2_ *d’*	–	n.s.	n.s.	–	n.s.	AD
	
		**Individual change over time in global task (slowdown at the second time)**			**Individual change over time in local task (slowdown at the second time)**		
	**Measure**	**Control**	**Congruent**	**Incongruent**	**Control**	**Congruent**	**Incongruent**
	
	RpTs	1 MCI/AD 2 AD	1 MCI/AD 1 AD	1 MCI 1 MCI/AD 1 AD	1 MCI/AD 3 AD	2 AD	1 MCI ^c^ 1 AD

		**Neuropsychological correlations with global visual processing > 0.50**			**Neuropsychological correlations with local visual processing > 0.50**		
**Neuropsy assessment in the longitudinal study**	**Measure**	**Control**	**Congruent**	**Incongruent**	**Control**	**Congruent**	**Incongruent**
	
	RpTs	Language^d,e^, executive functions^f,g,h,i,j,k^	L-V memory^l,m,n^, language^d,e,o^, executive functions^f,p,g,q,r,h,i,j,k^	Language^d^, executive functions^g,q,j,k^	Visual memory^s,n^, language^d,e^,executive functions^f,h^	Visual memory^n^^,^ executive functions^f,h^	Language^d,e^, executive functions^f,g,h,j^

RpTs, response times collected by the researcher; d’, discrimination sensitivity; Sp-Acc trade-off, speed-accuracy trade off; I_1–2_, *Change over time index*; HE, healthy elderly; MCI, mild cognitive impairment; MCI/AD, participants with MCI who progressed to probable AD; AD, Alzheimer’s disease; and n.s., not significant. ^a^Two participants, one belonging to the MCI group and the other to the AD group, were unable to identify any of the global figures presented. Thus, no global task was administered to these participants, but they did perform the local task. ^b^Slower responses were associated with worse discrimination, thus revealing a lack of speed-accuracy trade-off, in the congruent condition of the local task in the MCI and AD groups. ^c^Another participant with MCI performed the task more quickly in the 2nd moment of the assessment. ^d^Animal fluency. ^e^Names fluency. ^f^‘S’ fluency. ^g^Auto. Control latency. ^h^Digit span forward. ^i^Digit span backward. ^j^TMT B&W–A. ^k^TMT B&W–B. ^l^LM I: immediate. ^m^LM II: recognition. ^n^DMS: Set 2 abstract. ^o^Boston Naming Test. ^p^‘A’ fluency. ^q^Inhib. Control. ^r^Inhib. Hayling. ^s^DMS: Set 2 unique. Direction of the correlations can be consulted in Table 6.

## General discussion

### Changes in visual perception as possible early markers of Alzheimer’s disease progression

In the present article, we studied global/local visual processing and its evolution over time in healthy older adults and elderly people with MCI and AD. As dependent variables in our analyses, we used not only our participants’ RpTs but also the discrimination sensitivity (*d’*) of the Signal Detection Theory (Green and Swets, 1966), a measure of response quality that proved significantly useful to reveal the progressive attenuation of visual global precedence across healthy aging and Alzheimer’s disease. It should be noted that in contrast to previous studies, our results did not reveal a local advantage in either healthy (Oken et al., 1999; Lux et al., 2008; Lithfous et al., 2016) or cognitively impaired older adults (Coslett et al., 1995; Slavin et al., 2002; Blanca et al., 2010), but we did observe a bias toward priority processing of local information across MCI and AD. A plausible explanation for the absence of a clear local advantage in the present study is the randomized presentation of the stimuli in one of the four quadrants of the screen, given that the main studies that found a local bias in elderly participants conducted a central presentation of the stimuli (Slavin et al., 2002; Lux et al., 2008; Lithfous et al., 2016), a condition that has been shown to attenuate the GPE (Kimchi, 2015). Moreover, it is worth noting that the sample of older adults in the present study had a higher mean age than that reported in previous studies that also used hierarchical stimuli, being only comparable with the HE sample analyzed by Oken et al. (1999; *M* age = 83.6 years) who used geometric shapes as stimuli and, to a lesser extent with a sample of participants with AD studied by Slavin et al. (2002; *M* age = 78.5 years) who used numbers as stimuli. In all the other previous studies (Filoteo et al., 1992, 1995; Bruyer and Scailquin, 2000; Roux and Ceccaldi, 2001; Bruyer et al., 2003; Lux et al., 2008; Staudinger et al., 2011; Agnew et al., 2016; Lithfous et al., 2016; Bouhassoun et al., 2022), the mean age of the older adult samples ranged between 55 and 70 years. Thus, our results rule out the age of our participants as a determinant variable for the presence of a local bias in the present experiment, as they do reveal a differential pattern in each group of our older adults relative to their degree of cognitive impairment, both in their initial performance in the Navon letter perception task and in their progression after 1 year in the same task.

With regard to the results observed in typical elderly people, our HE group revealed the absence of global advantage and bidirectional interference in the cross-sectional study when RpTs were analyzed. However, we were able to demonstrate the presence of unidirectional global interference in this group of participants on examining the response quality (evaluated *via* discrimination sensitivity). Furthermore, the global/local processing profile was not altered after 1 year in this group of participants, none of whom reflected a significant change between the two assessment times in any of the analyzed measures. This finding offers evidence on the consistency of global/local visual processing in typical aging, characterized by the absence of neurodegenerative processes that could worsen performance over time.

In the cross-sectional study, the MCI group revealed both an absence of global advantage and bidirectional interference as well as a greater vulnerability than the HE group to the presence of any type of distractors in the first moment of assessment. Relevantly, this group, like the other two groups with cognitive impairment (MCI/AD and AD), showed behavioral changes after 1 year in the longitudinal measures analyzed. That is, *Change over time index* showed a better performance by the MCI group in the incongruent local task at the second moment. Also, a significant individual change was observed in 3 of the 5 group members, all the measures pointing to a lesser interference of global processing and a certain bias to prioritize local stimuli 1 year later. In addition, we would like to highlight that the four participants whose diagnosis varied from MCI to probable AD after 1 year were all women. This greater predisposition among women with MCI to develop more pronounced cognitive and functional impairments than men was already noted by Lin et al. (2015) and related to higher prevalence of the ApoE-e4 allele in women than in men (Farrer et al., 1997). It could then be assumed that the participants with MCI, as pointed out by Albert et al. (2011), are those who could be in the prodromal stage of Alzheimer’s dementia at the 1st moment of the assessment. In this regard, it is relevant to note that the participant with MCI who in the cross-sectional study was not able to perform the global task was one of the participants diagnosed with AD after 1 year, which reinforces the idea that the difficulty in forming global percepts by grouping local items could characterize prodromal stages of AD. Participants in the MCI/AD group also showed a more pronounced slowing after 1 year in the global task than in the local task, with one participant showing significant slowing in all the three conditions of the global task. The results of the longitudinal study, taken together for both the MCI and MCI/AD groups, showed that in the prodromal or early stages of AD slowing of RpTs was observed after 1 year, being more noticeable during the global than during the local analysis of the stimuli.

We should point out here that the only neuropsychological test that revealed differences between the MCI and MCI/AD groups was the number of correct scores on the Boston naming test, revealing that the participants in the MCI group whose diagnosis led to AD after 1 year showed language impairment. Thus, while there are authors who point out the usefulness of the Boston naming test 12-item version in differential diagnosis of AD with regard to HE (sensitivity 85%, specificity 94%, Serrano et al., 2001), our results suggest that poor performance on the Boston naming test might correlate with greater probability of developing AD from an MCI condition. Performance on this test has been related to activation of the left supramarginal gyrus, left superior temporal pole, right middle temporal gyrus, left inferior temporal gyrus, right inferior temporal gyrus, and left fusiform gyrus (Breining et al., 2022), suggesting that accentuated loss of gray matter in temporoparietal regions could be a risk factor for developing AD from an MCI condition.

Regarding the AD group, the participants revealed generalized slowing of their responses compared to the HE and MCI groups, as well as bidirectional interference in addition to the absence of global advantage in the cross-sectional study. Interestingly, when discrimination sensitivity was considered, this group discriminated letters better in the local than in the global task in the incongruent hierarchical stimuli, thus denoting, as suggested above, a lower sensitivity to the presence of global distractors. Furthermore, almost half of the participants in this group showed a relevant individual change after 1 year reflected by slowing down in different conditions of both the global and local tasks. Relevantly, significant results reflected by the ANOVAs with the RpTs indicated that AD displayed slower performance than HE in congruent trials of the global task and in control trials of both the global and local tasks. Thus, the longitudinal study revealed generalized slowing of processing speed in the AD group with a bias toward local analysis of stimuli. In fact, the only significant result regarding discrimination sensitivity was that AD carried out the incongruent local task better at the second moment of the assessment, showing enhanced priority processing of local information over time.

### Limitations of the present study and further directions of research

The present study has limitations that should be highlighted in order to advance in this line of research and, consequently, to expand the scope of the reported findings. On the one hand, we must mention the high mean age (∼87) of our participants and, thus, the high experimental mortality of the longitudinal study carried out. Although in the second study we have followed appropriate statistical procedures for analyzing results from small sample sizes, it would be advisable to carry out new longitudinal studies with larger sample sizes in order to replicate and expand the present findings. Additionally, due to both the very high mean age of our participants and their cognitive state, we had to reduce the trial’s structure, and the verbal responses were recorded by the experimenter. New studies should refine these solutions (e.g., with the use of microphones for response recording, by monitoring fixation with some system for online video imaging of the eye, with infrared-corneal reflection direction of gaze system or, alternatively, including a fixation point while reducing the excessive time to respond). In any event, it would be crucial to incorporate measures of performance other than RpTs. This is the case of discrimination sensitivity (*d’*) that we have used in the present study.

On the other hand, to our knowledge, this is the first study on global/local visual processing in elderly people with MCI. We have studied this group as a prodromal stage of dementia, which allows us to study the functioning of global/local visual processing through a continuum from the typical aging to the AD conditions. In this subject, Vogel et al. (2021), based on spatiotemporal trajectories of tau pathology, have proposed four AD subtypes, namely, ‘limbic,’ ‘posterior,’ ‘lateral temporal,’ and ‘medial-temporal lobe sparing,’ which present different cognitive profiles and differential disease progression. Future research might explore the implementation of factorial analyses that would include both neuropsychological and perceptual measures to investigate whether cognitive domains such as memory, language, and executive functions as well as global/local visual processing can constitute separate factors for defining a continuum of decline from mild cognitive impairment to dementia.

Additionally, in studies that start from genetic analysis of participants, it is relevant to study a possible differential performance in the Navon task between healthy elderly participants who have risk factors for developing AD and those who do not, in relation to performance in different neuropsychological tests. In this direction, Jacobson et al. (2005) conducted a prospective study with healthy older adults differentiating between those with and without the ApoE-e4 allele, as its presence is related to increased risk of developing AD. They showed that healthy older adults at higher risk for AD had more asymmetric performance in global and local learning (i.e., larger discrepancy between global/local learning scores) than older adults at lower risk for AD. Moreover, it would also be useful to complement the results obtained in the present study with electromagnetic brain tomography and functional neuroimaging techniques that allow us to establish the neurobiological correlates underlying the performance of global/local visual processing tasks. Using the event-related potentials technique, Lithfous et al. (2016) found that the P300 wave, related to the active grouping of the global stimulus, presented both shorter latency and greater amplitude in young than in older adults, as well as greater amplitude in global than local task only in young adults. It would be interesting to verify whether the MCI and AD groups also show changes in the mechanisms of active grouping of elements that could explain the differential pattern revealed during the processing of complex stimuli.

Finally, differences due to age and cognitive impairment in the global/local visual processing, as well as in the processing of other complex visual stimuli that require holistic processing such as faces (Tanaka and Gordon, 2011; Kurylo et al., 2017; Gerlach and Poirel, 2018; Gerlach and Starrfelt, 2018), define a developing line of research for their potential to differentiate between healthy older adults and those with AD, including the prodromal stage of the disease that constitutes MCI. In sum, assessing global/local visual processing along with other cognitive processes such as visual memory, executive functions, and language could allow us to establish possible markers of cognitive impairment and possible progression to Alzheimer’s dementia. Furthermore, early detection following a multidomain approach that includes measures of perceptual organization in the neuropsychological assessment is key to applying an intervention from early stages in the progression of dementia.

## Data availability statement

The datasets presented in this study can be found in online repositories. The names of the repository/repositories and accession number(s) can be found below: https://osf.io/2zp8a/.

## Ethics statement

The studies involving human participants were reviewed and approved by Ethics Committee of Universidad Autónoma de Madrid. The patients/participants provided their written informed consent to participate in this study.

## Author contributions

AA-SM: investigation, data curation, visualization, formal analysis, and writing (original draft). JI: conceptualization, project administration, funding acquisition, investigation, methodology, resources, writing (original draft and review and editing), and supervision. AG: methodology and formal analysis. EIO: conceptualization, project administration, funding acquisition, investigation, methodology, formal analysis, resources, writing (original draft and review and editing), and supervision. All authors read and approved the final version of the manuscript.

## Abbreviations

AD: Alzheimer’s disease
GPE: global precedence effect
HE: healthy elderly
MCI: mild cognitive impairment
RpTs: response times collected by the researcher.
