# Supplementary material for: Progressive attenuation of visual global precedence across healthy aging and Alzheimer’s disease
Source: Front Aging Neurosci. 2022 Sep 20;14:893818. doi: 10.3389/fnagi.2022.893818 (PMC9530062; doi:10.3389/fnagi.2022.893818)
Supplement: Supplementary file 1 [file Data_Sheet_1.pdf]

Supplementary_Material_Studies 1 & 2_Discrimination sensitivity

Section *2.2. Analyses of d'*

Table 2.2.a. Hit and false alarm (FA) probabilities for each group in the different experimental conditions in Study 1.

Group (*N*)	Task	Congruency	Hit prob.	FA prob.
HE *(26)*	Global	Congruent	.97 (.039)	.018 (.022)
		Incongruent	.87 (.22)	.043 (.071)
		Control	.99 (.001)	.016 (.022)
	Local	Congruent	.98 (.023)	.013 (.018)
		Incongruent	.82 (.27)	.10 (.20)
		Control	.96 (.07)	.025 (.048)
MCI *(21)*	Global	Congruent	.98 (.030)	.024 (.049)
		Incongruent	.84 (.29)	.14 (.29)
		Control	.99 (.001)	.068 (.19)
	Local	Congruent	.98 (.032)	.018 (.035)
		Incongruent	.76 (.33)	.080 (.12)
		Control	.96 (.051)	.024 (.042)
AD *(26)*	Global	Congruent	.91 (.20)	.038 (.057)
		Incongruent	.69 (.39)	.23 (.34)
		Control	.99 (.001)	.15 (.26)
	Local	Congruent	.95 (.10)	.039 (.081)
		Incongruent	.80 (.30)	.092 (.17)
		Control	.92 (.14)	.022 (.040)

Note: ±1.96·SD of the mean is shown in parentheses. HE (healthy elderly), MCI (Mild Cognitive Impairment), AD (Alzheimer's disease). Note that the control condition was not considered for calculating *d'*, however it did indicate an appropriate level of attention and ability of participants for the tasks [Mean Hit probability: .97 (range: .92-.99); Mean FA probability: .05 (range: .022-.15)].

Section ***5.2. Change over time index (I_{1-2})***

Table 5.2.b. Hit and false alarm (FA) probabilities for each group in the different experimental conditions in Study 2.

Group (*N*)	Task	Congruency	Hit prob.	FA prob.
HE *(9)*	Global	Congruent	.89 (.12)	.093 (.083)
		Incongruent	.78 (.25)	.18 (.26)
		Control	.93 (.082)	.12 (.11)
	Local	Congruent	.84 (.30)	.20 (.25)
		Incongruent	.78 (.26)	.17 (.30)
		Control	.83 (.32)	.16 (.29)
MCI *(5)*	Global	Congruent	.92 (.11)	.066 (.084)
		Incongruent	.79 (.17)	.25 (.32)
		Control	.90 (.13)	.13 (.21)
	Local	Congruent	.87 (.10)	.12 (.12)
		Incongruent	.83 (.15)	.22 (.31)
		Control	.90 (.087)	.19 (.24)
MCI/AD *(4)*	Global	Congruent	.96 (.066)	.049 (.078)
		Incongruent	.79 (.26)	.16 (.30)
		Control	.99 (.001)	.01 (.001)
	Local	Congruent	.99 (.001)	.001 (.001)
		Incongruent	.73 (.23)	.058 (.055)
		Control	.90 (.11)	.01 (.001)
AD *(11)*	Global	Congruent	.92 (.11)	.18 (.21)
		Incongruent	.48 (.37)	.41 (.36)
		Control	.91 (.11)	.24 (.32)
	Local	Congruent	.85 (.14)	.065 (.083)
		Incongruent	.93 (.11)	.088 (.086)
		Control	.89 (.15)	.089 (.17)

Note: ±1.96·SD of the mean is shown in parentheses. HE (healthy elderly), MCI (Mild Cognitive Impairment), MCI/AD (participants with MCI who progressed to probable AD), AD (Alzheimer's disease). Note that the control condition was not considered for calculating *d'*, however it did indicate an appropriate level of attention and ability of participants for the tasks [Mean Hit probability: .91 (range: .83-.99); Mean FA probability: .12 (range: .01-.24)].

Table 5.2.c. Estimated mean values and results of pairwise comparisons for *Group x Congruency x Task* comparing the I_{1-2} *d'* index between global and local tasks.

**Group**	**Congruency**	***M* Global**	***M* Local**	***F* (*df1*, *df2*)**	***p* (η^2)**
HE	Congruent	1.42 ± .78	2.53 ± .78	1.09 (1, 69)	.30 (.016)
	Incongruent	1.53 ± .78	1.43 ± .78	.009 (1, 69)	.93 (< .005)
MCI	Congruent	1.39 ±1.05	2.47 ±1.05	.58 (1, 69)	.44 (.008)
	Incongruent	1.39 ±1.95	- .012 ±1.05	.97 (1, 69)	.33 (.014)
MCI/AD	Congruent	1.12 ±1.35	- .38 ±1.17	.75 (1, 74)	.39 (.010)
	Incongruent	2.44 ±1.35	.34 ±1.17	1.48 (1, 74)	.23 (.20)
AD	Congruent	1.76 ± .74	1.48 ± .71	.08 (1, 71)	.78 (< .005)
	Incongruent	2.01 ± .78	-.48 ± .71	5.97 (1, 72)	**.018* (.076)**

Note: ±1.96·SE of the mean is shown under each value. *: Statistically significant differences (*p* < .05) are highlighted in bold. Note that a negative value reflects an improvement in task performance, whereas a positive value reflects a worsening after one year. HE (healthy elderly), MCI (Mild Cognitive Impairment), MCI/AD (participants with MCI who progressed to probable AD), AD (Alzheimer's disease).
