# Supplementary material for: Progressive attenuation of visual global precedence across healthy aging and Alzheimer’s disease
Source: Front Aging Neurosci. 2022 Sep 20;14:893818. doi: 10.3389/fnagi.2022.893818 (PMC9530062; doi:10.3389/fnagi.2022.893818)
Supplement: Supplementary file 2 [file Data_Sheet_2.pdf]

Supplementary_Material_Study 2_Neuropsychological assessment

Section *4.2. Materials and procedure*

Table 4.2.a. shows the cognitive domains and those indexes assessed for every administered task, means and standard errors for every group as well as results derived from both analyses and significant pair comparisons. No differences were found among groups in indexes such as Inhibitory Control, Digit Span Backward and TMT B&W (A and B versions). For the other indexes, the AD group showed poorer performance than the HE group. The AD group also performed more poorly than the MCI/AD group on several verbal memory (LM I: immediate, LM II: recognition, WL II: recognition) and visual memory (DMS48 set 1 unique, double and abstract errors and DMS48 set 2 unique errors) indices. Additionally, the AD group performed more poorly than the MCI group on indices of verbal (LM I: immediate and LM II: recognition) and visual (DMS48: Set 1 unique and double error) memory, language (Boston naming test) and executive functions ('S' verbal fluency and Hayling-Inhibitory part). Regarding the MCI/AD group, it performed more poorly than the HE group on the cognitive domains of verbal (LM I: immediate; WL II: delayed) and visual (DMS48: Set 2 unique) memory and language (Animal verbal fluency and Boston naming test), but performed more poorly than the MCI group solely on the Boston naming test (language). With regard to the MCI group, it performed more poorly than the HE group on the sub-indices LM I: immediate (verbal memory) and Animal verbal fluency (language).

Table 4.2.a. Means and standard error of group performance on every neuropsychological test.

Note: [a] Not all participants could perform all the tasks. [b]: Levene's test indicated unequal variances, so degrees of freedom were adjusted by Brown-Forsythe and a Games-Howell post-hoc pairwise comparison test was performed. [c] : The test for the equality of means could not be carried out for 'LM II: delayed' since the AD group presented a variance equal to 0. DV: dependent variables (analysed variables were those considered commonly to be more relevant for each task and condition). N: Number of participants included in the calculation of the mean, standard deviation.

*: Statistically significant differences ($p < .05$). The symbol '>' means better performance.

Cognitive domain	Neuropsychological test	HE Mean(SE)/N	MCI Mean(SE)/N	MCI/AD Mean(SE)/N	AD Mean(SE)/N	p (η^2)	Bonferroni
**Memory**	LM I: immediate (hits)	26 (2.0)/9	16.4 (2.29)/5	14.3 (2.69)/4	5.45 (1.27)/11	<.001* (.76)	HE > MCI, MCI/AD & AD<br>MCI & MCI/AD > AD
	LM II: delayed (hits) [c]	12.4 (2.62)/9	5.00 (2.43)/5	1.00 (1.41)/4	.00 (.00)/11	-	-
	LM II: recognition (hits) [b]	17.4 (.82)/9	14.0 (1.64)/5	10.8 (.95)/4	3.09 (1.60)/11	<.001* (.74)	HE, MCI & MCI/AD > AD
	WL I: immediate (hits)	18.1 (2.20)/9	12.4 (1.40)/5	11.0(1.41)/4	7.64 (1.44)/11	.001* (.47)	HE > AD
	WL II: delayed (hits) [b]	2.89 (.75)/9	1.60(.75)/5	.25 (.25)/4	.09 (.09)/11	.005* (.45)	HE > MCI/AD & AD
	WL II: recognition (hits)[b]	9.33 (.65)/9	8.60 (1.63)/5	9.75 (.85)/4	4.45 (1.48)/11	.010* (.32)	HE & MCI/AD > AD
	DMS: Set1 Unique (error)	1.13(.55)/8	3.25 (1.65)/4	2.25(.95)/4	7.67 (.76)/9	<.001* (.66)	HE, MCI & MCI/AD > AD
	DMS: Set1 Double (error)	2.13 (.69)/8	3.25 (.75)/4	3.50(1.19)/4	7.00 (.60)/9	<.001* (.59)	HE, MCI & MCI/AD > AD
	DMS: Set1 Abstract (error)	2.88 (.79)/8	4.25 (.85)/4	3.25(1.32)/4	8.00 (.71)/9	<.001* (.57)	HE & MCI/AD > AD
	DMS: Set2 Unique (error)[b]	.63 (.63)/8	4.50 (2.33)/4	3.25 (.48)/4	6.78 (.64)/9	.032* (.58)	HE > MCI/AD & AD<br>MCI/AD > AD
	DMS: Set2 Double (error)	3.25 (.92)/8	6.25 (1.80)/4	5.25 (.85)/4	7.67 (.58)/9	.002* (.42)	HE > AD
	DMS: Set2 Abstract (error)	2.00 (.60)/8	5.50 (1.94)/4	5.50 (.87)/4	5.24 (.65)/9	<.001* (.59)	HE > AD
**Language**	Animal fluency (hits)	16.8 (1.35)/9	9.80 (1.99)/5	7.25 (.95)/4	5.27 (.96)/11	<.001* (.68)	HE > MCI, MCI/AD & AD
	Names fluency (hits)	18.2 (1.82)/9	12.0 (2.30)/5	11 (1.47)/4	7.27 (1.25)/11	<.001* (.52)	HE > AD
	Boston naming test (hits)	9.33 (.78)/9	10.0 (.89)/5	5.00 (1.23)/4	2.73 (.83)/11	<.001* (.66)	HE > MCI/AD & AD<br>MCI > MCI/AD & AD
**Executive functions**	'S' fluency (hits)	12.4 (2.31)/8	10.0 (1.29)/4	5.00 (1.41)/4	2.27 (.87)/11	<.001* (.55)	HE > AD<br>MCI > AD
	'A' fluency (hits)	10.1 (2.51)/8	8.75 (1.32)/4	4.00 (1.47)/4	2.64 (.99)/11	.011* (.38)	HE > AD
	Auto. Control latency (RTs)	19.9 (1.36)/9	25.6 (5.19)/5	27.5 (3.33)/4	36.9 (6.27)/7	.041* (.32)	HE > AD
	Inhib. Control (error)	5.44 (2.51)/9	2.40 (1.60)/5	8.00 (4.02)/4	9.14 (3.77)/7	.50	-
	Auto. Hayling latency (RTs)	18.2 (1.71)/9	21.0 (3.35)/5	25.3 (7.57)/4	62.4 (14.0)/10	.009* (.38)	HE > AD
	Inhib. Hayling (error)	13.9 (3.88)/9	16.0 (8.78)/5	29.0 (5.61)/4	33.6 (3.18)/10	<.002* (.46)	HE & MCI > AD
	Digit span Forward (hits)	9.11 (.79)/9	6.00 (.89)/5	6.00 (.82)/4	5.91 (.63)/11	.010* (.36)	HE > AD
	Digit span Backward (hits)	6.33 (.58)/9	5.00 (.71)/5	4.75 (.50)/4	4.36 (.49)/11	.065	-
	TMT B&W – A (RTs)	137 (24.0)/9	218 (53.4)/5	305 (45.3)/4	275 (73.7)/4	.047* (.35)	n.s.
	TMT B&W – B (RTs)	331 (56.2)/9	463 (117)/4	615 (191)/3	481 (88.5)/3	.24	-
