# Supplementary material for: Progressive attenuation of visual global precedence across healthy aging and Alzheimer’s disease
Source: Front Aging Neurosci. 2022 Sep 20;14:893818. doi: 10.3389/fnagi.2022.893818 (PMC9530062; doi:10.3389/fnagi.2022.893818)
Supplement: Supplementary file 3 [file Data_Sheet_3.pdf]

**Supplementary_Material_Study 2_Response times**

**Section *5.2. Change over time index ($I_{1\text{-}2}$)***

Table 5.2.a. Estimated mean values and results of pairwise comparisons for *Group x Congruency x Task* comparing the $I_{1\text{-}2}$ RpTs index between global and local tasks.

| Group | Congruency | *M* Global | *M* Local | *F* (*df1*, *df2*) | *p* ($\eta p^2$) |
|---|---|---|---|---|---|
| HE | Control | -122 ±79.6 | -70.5 ±79.6 | .29 (1, 118) | .59 |
| | Congruent | -64.7 ±79.6 | -73.1 ±79.6 | .008 (1, 118) | .93 |
| | Incongruent | -116 ±79.6 | -85.1 ±79.6 | .11 (1, 118) | .74 |
| MCI | Control | -52.6 ±107 | -36.0 ±107 | .017 (1, 118) | .90 |
| | Congruent | -132 ±107 | -33.6 ±107 | .61 (1, 118) | .44 |
| | Incongruent | -427 ±107 | -8.40 ±107 | 10.9 (1, 118) | **.001 * (.085)** |
| MCI/AD | Control | -198 ±135 | -117 ±119 | .27 (1, 122) | .60 |
| | Congruent | -73.8 ±135 | -131 ±119 | .14 (1, 122) | .71 |
| | Incongruent | -339 ±135 | -18.4 ±119 | 4.27 (1, 122) | **.041 * (.034)** |
| AD | Control | -124 ±74.9 | -137 ±72 | .023 (1, 120) | .88 |
| | Congruent | -75.8 ±74.9 | -151 ±72.0 | .73 (1, 120) | .40 |
| | Incongruent | -160 ±78.2 | -12.0 ±72.0 | 2.66 (1, 120) | .11 |

*Note*: ±1.96·SE of the mean is shown under each value. *: Statistically significant differences (*p* < .05) are highlighted in bold. Note that a negative value reflects a slowdown in the task whereas a positive value reflects an acceleration of RpTs after one year. HE (healthy elderly), MCI (Mild Cognitive Impairment), MCI/AD (participants with MCI who progressed to probable AD), AD (Alzheimer's disease).

**Section *5.3. Individual change in participants over time***

Table 5.3.a. Number of participants from each studied group presenting a significant change in their RpTs one year on from the 1st assessment.

| **Group (*N*)** | **Global control** | **Global congruent** | **Global incongruent** | **Local control** | **Local congruent** | **Local incongruent** |
|---|---|---|---|---|---|---|
| HE *(9)* | 0 | 0 | 0 | 0 | 0 | 0 |
| MCI *(5)* | 0 | 0 | 1 | 0 | 0 | 2* |
| MCI/AD *(4)* | 1 | 1 | 1 | 1 | 0 | 0 |
| AD *(11)* | 2 | 1 | 1 | 3 | 2 | 1 |

*Note*: *All participants presented a slowdown of their RpTs after one year, excepting one participant with MCI in the incongruent condition of the local task, who performed the task more quickly in the 2nd moment of assessment. HE (healthy elderly), MCI (Mild Cognitive Impairment), MCI/AD (participants with MCI who progressed to probable AD), AD (Alzheimer's disease).
